# Sex-Specific Differences in Visceral and Subcutaneous Adiposity Accumulation and Their Association With Metabolic Abnormalities

**DOI:** 10.1155/jobe/7240063

**Published:** 2025-10-27

**Authors:** Isa Galvão Rodrigues, Gabriela Maria Pereira Floro Arcoverde, Camila Lima Chagas do Nascimento, Victoria Domingues Ferraz, Nadja Fernandes da Silva, Ilma Kruze Grande de Arruda, Cláudia Porto Sabino Pinho, Dário Celestino Sobral Filho

**Affiliations:** ^1^Department of Nutrition, Emergency Cardiology Unit of the University of Pernambuco, Recife, State of Pernambuco, Brazil; ^2^Faculty of Medical Sciences, University of Pernambuco, Recife, State of Pernambuco, Brazil; ^3^Department of Nutrition, Federal University of Pernambuco, Recife, State of Pernambuco, Brazil; ^4^Department of Nutrition, Clinics Hospital, Federal University of Pernambuco, Recife, State of Pernambuco, Brazil

**Keywords:** abdominal obesity, cardiometabolic risk factors, intra-abdominal fat, sex

## Abstract

Some studies suggest that body fat distribution differs between sexes; there remains a gap regarding the exact mechanisms that regulate these differences and their cardiometabolic consequences. This study investigated sex-specific differences influencing the concentration and metabolic risk associated with visceral abdominal adipose tissue (VAT) and subcutaneous adipose tissue (SAT). This cross-sectional study includes outpatients from a university-affiliated public hospital. Consecutive patients aged ≥ 20 years were included in our sample. VAT and SAT were measured using ultrasound (the mean of three attempts). Intra- and interevaluator reproducibility was tested, demonstrating high reliability (> 0.90) for both VAT and SAT. Demographic (age, sex, and self-reported race), anthropometric, behavioral, and biochemical variables were assessed. A total of 253 patients were included. They had a mean age of 46.3 ± 11.6 years (64.4% females and 68.7% non-white). Although the mean total abdominal adipose tissue was similar between sexes (*p*=0.125), males exhibited a higher mean VAT (7.3 ± 3.0 vs. 6.0 ± 2.1 cm; *p* < 0.001), while women presented with a higher mean SAT (3.4 ± 1.2 vs. 2.7 ± 1.4 cm; *p* < 0.001). Among females, VAT was directly associated with serum triglycerides (TG), TG/HDL ratio, blood glucose, and glycated hemoglobin (HbA1c), and inversely associated with HDL. VAT/SAT ratio predicted higher levels of total cholesterol (TC), LDL, TG, TG/HDL ratio, glucose, and HbA1c (*p* < 0.05). Among males, VAT did not significantly affect metabolic alterations. In conclusion, within the same mean BMI, males had higher VAT concentrations, whereas in females, despite lower VAT, a worse adverse metabolic profile was observed.

## 1. Introduction

Over the past 3 decades, the rising prevalence of overweight and obesity has been described as a global pandemic, closely linked to increasing rates of obesity-related disorders such as hypertension, dyslipidemia, and diabetes mellitus [1, 2]. Abdominal fat accumulation may be the most significant risk factor for several morbid conditions, which may occur even in the absence of generalized overweight or obesity [2]. This abdominal fat includes subcutaneous and intra-abdominal fat depots, the latter of which is divided into visceral (intraperitoneal) and retroperitoneal fat [3].

Excess visceral adipose tissue (VAT) is particularly associated with metabolic dysregulation, exhibiting proatherogenic and pro-inflammatory properties, being identified as a major risk factor for cardiovascular morbidity and mortality [3–5]. Subcutaneous adipose tissue (SAT) has also been linked to an increased risk of cardiovascular disease, though its effects are less harmful and not always consistent [6]. Since absolute measurements of adipose tissue compartments may not reflect overall body fat distribution but instead represent unique fat deposits, the VAT/SAT ratio has been suggested as a better indicator of an individual's predisposition to visceral fat accumulation.

Previous studies have shown that a higher VAT/SAT ratio strongly predicts carotid artery plaque presence, also being an independent predictor of mortality and coronary events [7–9]. Even though some studies suggest that body fat distribution differs between sexes [10], there remains a gap regarding the exact mechanisms that regulate these differences and their cardiometabolic consequences. In this study, we investigated sex-specific differences that influence the concentration and metabolic risk associated with VAT and SAT compartments.

## 2. Methods

### 2.1. Study Design and Sample Size

This cross-sectional study involved consecutive outpatients from a university-affiliated public hospital specializing in cardiology, located in Northeast Brazil. This study's protocols adhered to ethical guidelines for research involving human subjects, having been approved by the Research Ethics Committee of the HUOC/PROCAPE Hospital Complex, under protocol number 44488521.5.0000.5192. All participants provided written informed consent. Males and females aged ≥ 20 years were included, and recruited through voluntary participation. Exclusion criteria were pregnant women, individuals with concomitant consumptive diseases (e.g., nephropathies, cancer, or congestive heart failure grades III and IV, and severe infections), those with hypo- or hyperthyroidism, those undergoing pharmacological therapy for weight loss, hormone therapy, or lipid-lowering medications, those with cognitive deficits or psychological issues, and individuals with physical limitations that prevented the collection of anthropometric measurements.

Assuming a 5% α error, a β error of 20%, an estimated average correlation between the VAT and the metabolic parameters of 0.5 (p) [11], and a variability of 0.1 (*d*^2^), it was obtained a minimum sample size of 197 individuals. To correct possible losses, that number was increased by 30%, with a total sample of *n* = 257.

### 2.2. Ultrasound (US) Measurements

VAT and SAT measurements were assessed using the Apogee 3500 Digital Color Doppler Ultrasound Imaging System (SIUI, Shantou, China), with a convex transducer at a frequency of 4.0 MHz, following the protocol proposed by Mauad et al. [12]. Participants fasted for at least 4 h, were positioned in a supine position with their right arm elevated. The transducer was placed transversely 2 cm above the umbilical scar, without applying pressure on the abdomen to avoid underestimation. VAT was measured as the distance in centimeters from the internal (deep) surface of the rectus abdominis muscle to the anterior wall of the aorta, with the individual in expiration. SAT was measured as the distance between the external fascia of the rectus abdominis muscle and the skin's lower boundary [12, 13]. The VAT/SAT ratio was calculated by dividing the absolute VAT value by the SAT value. Total abdominal adipose tissue (TAT) was determined by summing VAT and SAT, as described by Grunnet et al. [14]. Before US data collection, evaluators underwent training, and the technique was standardized. All measurements were conducted three times, and the mean value was utilized. Intra- and interevaluator reproducibility were tested. Intraclass Correlation Coefficient (ICC) was determined, with 95% limits of agreement [13].

### 2.3. Co-Variates

Demographic (age, sex, and self-reported race), anthropometric, behavioral, and biochemical variables were assessed. Anthropometric parameters included body mass index (BMI), computed as weight (kg)/height (m)^2^, and waist circumference (WC), classified according to the guidelines proposed by the World Health Organization [15]. Measurements were also conducted three times, and mean values were utilized for analyses. Weight (kg) and height (m) were measured following the techniques recommended by Lohman et al. [16]. WC was measured using a nonflexible tape measure with an accuracy of 0.1 cm, directly on the skin, during expiration, with the individual standing, abdomen relaxed, arms alongside the body, and feet together, at the narrowest point between the chest and the hips [16].

Lifestyle/behavioral variables included alcohol consumption and physical activity. Alcohol consumption was assessed by asking participants whether they had consumed alcoholic beverages in the 30 days before the questionnaire, with a dichotomous yes/no response [13]. Physical activity levels were determined using the short version of the International Physical Activity Questionnaire [17], which considers four domains: leisure, household activities, occupational activities, and transport-related activities. Individuals engaging less than 150 min/week were classified as insufficiently active or sedentary [18].

### 2.4. Metabolic Biomarkers

Metabolic biomarkers were measured in blood samples collected after 12 h of fasting. The following parameters were assessed: fasting glucose, glycated hemoglobin (HbA1C in %), lipid profile (triglycerides [TG], total cholesterol [TC], and fractions), and the TG/HDL ratio, calculated by dividing the absolute TG value by the HDL value.

### 2.5. Data Analysis

Data were analyzed using the Statistical Package for Social Sciences (SPSS), Version 13.0 (IBM Inc., Chicago, IL, USA). Exploratory data analysis was performed, and outliers (±3 standard deviations from the mean) were excluded (*n* = 3). Continuous variables were tested for normality using the Kolmogorov–Smirnov test. Data from normally distributed variables were expressed as mean ± standard deviation. Variables with non-Gaussian distributions underwent logarithmic (Ln) transformation and were retested for normality; as they remained non-normally distributed, they were described as medians and interquartile ranges. For categorical variables, the binomial distribution was approximated to the normal distribution using a 95% CI. The student's “*t*” test for independent samples was used to compare variables between sexes. Proportions were compared using Pearson's chi-square test or Fisher's exact test. Simple linear regression was applied to assess the explanatory power of VAT concerning biochemical parameters. Intra- and interevaluator reproducibility was determined using the ICC, with the following classification: high or excellent agreement (ICC > 0.75), satisfactory agreement (ICC between 0.4 and 0.75), and poor agreement (ICC < 0.4) [18]. Statistical significance was set at *p* < 0.05 for all analyses.

## 3. Results

Intra- and interevaluator reproducibility for US measurements (i.e., VAT and SAT) demonstrated high intraevaluator reproducibility: ICC > 0.97 for VAT and > 0.98 for SAT. Interevaluator reproducibility was similarly high: ICC > 0.90 for all VAT and SAT measurements. Regarding our sample, 290 patients were initially eligible and recruited, but 37 had no biochemical test results, leaving a final sample of 253 patients. The mean age was 46.3 ± 11.6 years, with a higher proportion of females (64.4%) and non-white individuals (68.7%). A total of 45.8% of individuals reported alcohol consumption, and 75.8% were insufficiently active. Overweight was found in 60.1% of the sample, and elevated WC was found in 64.1% (Table 1).

Males were older and had a higher proportion of hypertension and diabetes compared to females (*p* < 0.05). Although TAT was similar between sexes (*p*=0.125), men had a higher VAT (7.3 ± 3.0 vs. 6.0 ± 2.1 cm; *p* < 0.001), while women had a higher SAT (3.4 ± 1.2 vs. 2.7 ± 1.4 cm; *p* < 0.001). VAT/SAT ratios were higher in men (*p* < 0.001) (Table 1). In men, VAT and the VAT/SAT ratio were direct predictors of changes in glycated hemoglobin and triglyceride levels. SAT was not a predictor of metabolic changes in men and was inversely related to glycated hemoglobin levels (*p*=0.001). For each 1 cm increase in VAT, glucose levels increased by 3.5 mg/dL (*p*=0.052; *r*^2^: 4.3%) (Table 2).

In women, VAT and the VAT/SAT ratio were predictors of several metabolic changes. VAT was directly related to triglyceride levels, TG/HDL ratio, glucose, and glycated hemoglobin, and inversely related to HDL. A higher VAT/SAT ratio predicted elevated levels of TC, LDL, triglycerides, the TG/HDL ratio, glucose, and glycated hemoglobin (*p* < 0.05). SAT, on the other hand, was inversely related to TC levels (*p* < 0.05) (Table 3).

## 4. Discussion

This study investigated sex-specific differences that modulate the metabolic risk associated with excess VAT and SAT, demonstrating the following key findings: (1) Men are more predisposed to accumulate VAT, while women tend to have a higher concentration of SAT; (2) VAT measurements (either alone or through the VAT/SAT ratio) US-derived demonstrated good predictive ability for determining metabolic risk in women, while in men, these measurements did not appear to be sufficiently accurate.

Previous studies have also demonstrated a higher concentration of VAT in men [19, 20]. In one study examining sex-related differences in the contribution of US-derived VAT and SAT to the fatty liver index in overweight and obese Caucasian adults, men had a mean VAT of 7.7 cm, compared to 4.9 cm in women [19]. Similarly, Bertoli et al. [20] found higher VAT values assessed by US in men (mean of 7.1 cm) compared to women (mean of 4.2 cm) in a study of Italian patients aged 18–66 years [20]. However, Hazem et al. [7] reported different results: they found lower VAT values in men (6.38 cm vs. 7.15 cm in women) [7]. These varying findings highlight the importance of continuing research on this topic, especially across different ethnic groups, to explore the full spectrum of obesity phenotypes and sex-specific factors.

Previous studies also reinforce the greater tendency for women to have a higher concentration of SAT [7, 21, 22]. Notably, there are significant differences between men and women in fat distribution, as well as the properties of adipocytes within anatomical fat deposits, partially explained by hormonal differences [23]. Although the specific role of each hormone in modulating body fat is not fully understood, evidence suggests that a relatively higher amount of estrogen and progesterone receptors, compared to androgen receptors, contributes to fat storage in the glute-femoral subcutaneous region but not in VAT. In contrast, visceral fat contains higher levels of androgen receptors, which may explain the greater accumulation of visceral fat in men [24].

Genetic features may also explain the sex differences in adipose tissue concentration and distribution, as sex-specific genetic determinants for fat accumulation have been identified [22]. In a genome-wide association study on visceral and subcutaneous fat, Fox et al. [25] identified 12 single nucleotide polymorphisms (SNPs) associated with VAT in women and only 2 SNPs in men. These findings highlight the influence of genetic components on body composition and underscore the need for further research to identify the genetic patterns that contribute to an unfavorable adiposity profile. It is relevant to note that our sample was homogeneous regarding many characteristics, such as race, physical activity level, BMI, and alcohol consumption.

In addition to the sex differences in VAT and SAT concentrations, our results demonstrated differences in the metabolic risk associated with VAT and the VAT/SAT ratio. In women, both parameters effectively captured adverse metabolic risk, being related to 5 (HDL-c, TG, TG/HDL-c ratio, glucose, and glycated hemoglobin) and 6 (TC, LDL-c, TG, TG/HDL-c ratio, glucose, and glycated hemoglobin) of the 7 metabolic parameters assessed, respectively. In contrast, in men, VAT was associated with only 2 metabolic parameters (TG and glycated hemoglobin). The greater predictive capacity of VAT for identifying metabolic alterations in women has also been demonstrated by other authors [6, 22, 26]. These findings underscore the potential morphological and functional differences in adipose tissue between men and women, with distinct responses to the metabolic risk associated with ectopic fat accumulation. However, few explanations have been proposed to clarify these disparities.

Despite the more unfavorable scenario for males, including a higher proportion of individuals with hypertension, DM and older age, the VAT could not capture metabolic changes, and it is possible that other factors explain the greater attributable risk in men. In any case, men notably accumulate a greater concentration of muscle mass, which can be a potent protective factor [27–29]. Therefore, analyzing the impact of a single body composition factor on cardiometabolic risk in isolation can limit possible interpretations, making it essential to investigate the synergy and interaction between risk factors and protective factors, which can offset the deleterious effects.

It is also important to consider that SAT is structurally heterogeneous, being divided into superficial and deep SAT. In obese individuals, deep SAT expands more and exhibits biological characteristics similar to VAT, being associated with increased inflammatory activity and oxidative stress [30]. Although our study did not assess SAT subcompartments, it is plausible to theorize that higher abdominal SAT observed in women may include the deep compartment, contributing to an increased metabolic risk [31].

Sex-specific differences demonstrated in this study highlight the need for sex-specific cutoff points for cardiometabolic risk attributed to VAT. Our findings indicate that, for the same average BMI, men had a higher concentration of VAT, illustrating the limitation of BMI in capturing sex differences in intra-abdominal fat accumulation. At the same time, women, despite having lower VAT concentrations, exhibited a worse adverse metabolic profile associated with excess VAT. Therefore, it is essential to adopt distinct cutoff points for men and women when evaluating VAT as a screening tool for metabolic risk.

Limitations of this study include potential selection bias and may have limited generalizability, as the study population was conducted in a single center. The cross-sectional design additionally precludes causal interpretations. Longitudinal studies are needed to determine whether the sex-specific differences translate into a higher incidence of cardiometabolic disorders/events. Moreover, the absence of relevant variables, such as diet and genetic variations, limits the contextualization of the issue. It is well known that diet assessment is complex and remains a gap in many studies on this topic. Additionally, men had a higher average age and a greater percentage of individuals with diabetes and hypertension, factors that may be related to the higher concentration of VAT observed in this group. Therefore, sex differences in this study should be interpreted with caution, and future investigations should consider adjusted comparative analyses.

Strengths of our study include the racial characteristics of our population. While studies have shown sex differences in VAT and SAT among Caucasians and Asians, these differences have not yet been demonstrated in other ethnic groups. Our population, being racially mixed, adds to the growing body of evidence. Furthermore, we used standardized techniques and maintained methodological rigor in obtaining US measurements, achieving high intraobserver and interobserver reproducibility.

## 5. Conclusion

This study demonstrates substantial sex-specific differences in VAT and SAT deposits, with men showing a higher concentration of VAT and women having a higher concentration of SAT. Despite having lower VAT deposits, VAT is a strong predictor of metabolic risk in women. This predictive capacity was not observed among men. Our study emphasizes the need to better understand the influence of sex on abdominal fat accumulation and the development of metabolic dysfunctions across different ethnic groups to implement more personalized approaches for the prevention or treatment of this condition. Future studies with longitudinal designs could help clarify how these differences translate into incident events. Additionally, investigating different body segments, such as muscle mass, may aid in understanding the body phenotype that confers a higher metabolic risk.

## Figures and Tables

**Table 1:** Patients' characteristics (*N* = 253).

**Variables**	**Total**	**Males (*N* = 90)**	**Females (*N* = 163)**	**p**valu**e**^∗^
	***N* (%)**	***N* (%)**	***N* (%)**	

Age categories (years)				< 0.001
< 60	225 (88.9)	71 (78.9)	154 (94.5)	
≥ 60	28 (11.1)	19 (21.1)	9 (5.5)	
Ethnicity				0.324
White	79 (31.2)	23 (25.6)	56 (34.4)	
Black	55 (21.7)	20 (22.2)	35 (21.5)	
Mixed	119 (47.0)	47 (52.2)	72 (44.2)	
HTN				< 0.001
No	145 (57.3)	34 (37.8)	111 (68.1)	
Yes	108 (42.7)	56 (62.2)	52 (31.9)	
DM				< 0.001
No	203 (80.2)	58 (64.4)	145 (89.0)	
Yes	50 (19.8)	32 (35.6)	18 (11.0)	
Alcohol intake				0.846
No	137 (54.2)	48 (53.3)	89 (54.6)	
Yes	116 (45.8)	42 (46.7)	74 (45.4)	
Physical activity status				0.124
< 150 min/week	188 (75.8)	26 (29.9)	34 (21.1)	
≥ 150 min/week	60 (24.2)	61 (70.1)	127 (78.9)	
BMI categories				0.157
Underweight	38 (15.0)	10 (11.0)	28 (17.2)	
Normal range	63 (24.9)	28 (31.1)	35 (21.5)	
Excess weight	152 (60.1)	52 (57.8)	100 (61.3)	
WC categories				0.004
Normal	90 (35.9)	42 (47.7)	48 (29.4)	
Elevated	161 (64.1)	46 (52.3)	115 (70.6)	

**Variables**	**Mean (SD) or median (IR)**	**Mean (SD) or median (IR)**	**Mean (SD) or median (IR)**	**p** **value**

Age (years old)	46.3 ± 11.6	50.4 ± 12.8	44.0 ± 10.3	< 0.001^a^
BMI (kg/m^2^)	27.2 ± 7.4	27.1 ± 6.9	27.3 ± 7.7	0.794^a^
WC (cm)	90.3 ± 14.4	95.1 ± 15.5	87.7 ± 13.1	< 0.001^a^
TC (mg/dL)	177.6 ± 53.2	177.3 ± 51.3	177.8 ± 54.4	0.937^a^
HDL (mg/dL)	45.3 ± 12.3	40.1 ± 11.1	48.1 ± 12.1	< 0.001^a^
LDL (mg/dL)	111.9 ± 38.9	102.6 ± 44.7	117.0 ± 34.5	0.010^a^
TG (mg/dL)	109.0 (82.0–151.4)	130.0 (90.9–166.0)	103.0 (78.0–141.7)	0.002^b^
Glucose (mg/dL)	94.9 (88.0–104.5)	97.0 (88.2–121.5)	94.0 (88.0–99.0)	0.008^b^
HbA1C (%)	5.6 (5.3–6.2)	5.78 (5.3–6.65)	5.5 (5.3–6.0)	0.043^b^
VAT (cm)	6.5 ± 2.5	7.3 ± 3.0	6.0 ± 2.1	< 0.001^a^
SAT (cm)	3.2 ± 1.3	2.7 ± 1.4	3.4 ± 1.2	< 0.001^a^
TAT (cm)	9.6 ± 2.9	10.0 ± 3.3	9.4 ± 2.7	0.125^a^
VAT/SAT ratio	1.8 (1.5–2.8)	2.7 (1.8–4.2)	1.8 (1.4–2.2)	< 0.001^b^
VAT/TAT ratio	0.7 ± 0.1	0.7 ± 01	0.6 ± 0.1	< 0.001^a^

Abbreviations: BMI, body mass index; DM, diabetes mellitus; HbA1c, glycated hemoglobin; HDL, high-density lipoprotein; HTN, hypertension; LDL, low-density lipoprotein; SAT, subcutaneous adipose tissue; TC, total cholesterol; TG: triglycerides; VAT, visceral adipose tissue; WC, waist circumference.

^∗^Pearson's chi-square.

^a^T de Student's test for comparing means.

^b^U de Mann–Whitney test for comparing medians.

**Table 2:** VAT, SAT, and VAT/SAT ratio as predictors of metabolic alterations in men (*n* = 90).

Metabolic biomarkers	*B*	EP	ß	*p* ^a^	R^2b^
*VAT*					
TC (mg/dL)	2.038	1.830	0.118	0.268	1.4
HDL (mg/dL)	−0.360	0.395	−0.097	0.365	0.9
LDL (mg/dL)	1.364	1.678	0.087	0.419	0.8
TG (mg/dL)	11.576	5.211	0.230	0.029	5.3
TG/HDL	0.487	0.352	0.146	0.170	2.1
Glucose (mg/dL)	3.579	1.816	0.207	0.052	4.3
HbA1C (%)	0.208	0.053	0.405	< 0.001	16.4

*SAT*					
TC (mg/dL)	0.031	3.804	0.001	0.994	1.0
HDL (mg/dL)	−0.694	0.817	−0.090	0.398	0.8
LDL (mg/dL)	−0.090	3.347	−0.003	0.979	0
TG (mg/dL)	10.920	10.992	−0.105	0.322	1.1
TG/HDL	−0.558	0.732	−0.081	0.447	0.7
Glucose (mg/dL)	−3.494	3.810	−0.098	0.362	1.0
HbA1C (%)	−0.372	0.111	−0.351	0.001	12.3

*VAT/SAT ratio*					
TC (mg/dL)	1.023	1.239	0.088	0.412	0.8
HDL (mg/dL)	0.352	0.266	0.140	0.189	2.0
LDL (mg/dL)	0.672	1.216	0.059	0.582	0.4
TG (mg/dL)	7.277	3.521	0.215	0.042	4.6
TG/HDL	0.204	0.239	0.091	0.395	0.8
Glucose (mg/dL)	2.277	1.228	0.195	0.067	3.8
HbA1C (%)	0.174	0.033	0.507	< 0.001	25.7

*Note:* Linear regression analysis.

Abbreviations: HbA1c, glycated hemoglobin; HDL, high-density lipoprotein, HTN, Hypertension; LDL, low-density lipoprotein; SAT, subcutaneous adipose tissue; SE, standardized error; TC, total cholesterol; TG, triglycerides; VAT, visceral adipose tissue.

^a^ *p* value.

^b^coefficient of determination.

**Table 3:** VAT, SAT, and VAT/SAT ratio as predictors of metabolic alterations in women (*n* = 163).

Metabolic biomarkers	*B*	SE	ß	*P*	*R* ^2^
*VAT*					
TC (mg/dL)	1.346	2.075	0.051	0.517	0.3
HDL (mg/dL)	−1.137	0.453	−0.195	0.013	3.8
LDL (mg/dL)	1.492	1.312	0.089	0.257	0.8
TG (mg/dL)	8.816	1.956	0.335	< 0.001	11.2
TG/HDL	0.243	0.052	0.345	< 0.001	11.9
Glucose (mg/dL)	3.662	1.317	0.214	0.006	4.6
HbA1C (%)	0.171	0.052	0.264	0.001	7.0

*SAT*					
TC (mg/dL)	−6.867	3.365	−0.159	0.043	2.5
HDL (mg/dL)	−0.440	0.759	−0.046	0.563	0.2
LDL (mg/dL)	−0.823	2.166	−0.030	0.705	0.1
TG (mg/dL)	5.192	3.380	0.120	0.126	1.4
TG/HDL	0.141	0.091	0.122	0.123	1.5
Glucose (mg/dL)	−4.158	2.187	−0.148	0.059	2.2
HbA1C (%)	−0.153	0.089	−0.141	0.087	2.0

*VAT/SAT ratio*					
TC (mg/dL)	13.152	4.122	0.209	0.008	4.3
HDL (mg/dL)	−0.521	0.938	−0.044	0.579	0.2
LDL (mg/dL)	5.332	2.645	0.157	0.045	2.5
TG (mg/dL)	10.863	4.122	0.203	0.009	4.1
TG/HDL	0.221	0.112	0.155	0.050	2.4
Glucose (mg/dL)	10.769	2.600	0.310	< 0.001	9.6
HbA1C (%)	0.471	0.101	0.361	< 0.001	13.1

*Note:* Linear regression analysis.

Abbreviations: HbA1c, glycated hemoglobin; HDL, high-density lipoprotein; HTN, hypertension; LDL, low-density lipoprotein; SAT, subcutaneous adipose tissue; SE, standardized error; TC, total cholesterol; TG, triglycerides; VAT, visceral adipose tissue.

## Data Availability

The datasets generated during the current study are available from the corresponding author.
